# Peripheral Nerve-Derived Stem Cell Spheroids Induce Functional Recovery and Repair after Spinal Cord Injury in Rodents

**DOI:** 10.3390/ijms22084141

**Published:** 2021-04-16

**Authors:** Hye-Lan Lee, Chung-Eun Yeum, HyeYeong Lee, Jinsoo Oh, Jong-Tae Kim, Won-Jin Lee, Yoon Ha, Young-Il Yang, Keung-Nyun Kim

**Affiliations:** 1 Spine & Spinal Cord Institute, Department of Neurosurgery, College of Medicine, Yonsei University, Seoul 03722, Korea; hllee4@yuhs.ac (H.-L.L.); kimura416@yuhs.ac (H.L.); 187OJS@yuhs.ac (J.O.); HAYOON@yuhs.ac (Y.H.); 2 Paik Inje Memorial Institute for Clinical Research, Inje University College of Medicine, Busan 47392, Korea; perfectgirl0806@gmail.com (C.-E.Y.); jtfreejuly@gmail.com (J.-T.K.); wonjin257@gmail.com (W.-J.L.); 3 POSTECH Biotech Center, Pohang University of Science and Technology (POSTECH), Pohang, Gyeongbuk 37673, Korea

**Keywords:** peripheral nerve-derived stem cells, spheroids, spinal cord injury, neuroregeneration, functional recovery, neurotrophic factor

## Abstract

Stem cell therapy is one of the most promising candidate treatments for spinal cord injury. Research has shown optimistic results for this therapy, but clinical limitations remain, including poor viability, engraftment, and differentiation. Here, we isolated novel peripheral nerve-derived stem cells (PNSCs) from adult peripheral nerves with similar characteristics to neural-crest stem cells. These PNSCs expressed neural-crest specific markers and showed multilineage differentiation potential into Schwann cells, neuroglia, neurons, and mesodermal cells. In addition, PNSCs showed therapeutic potential by releasing the neurotrophic factors, including glial cell-line-derived neurotrophic factor, insulin-like growth factor, nerve growth factor, and neurotrophin-3. PNSC abilities were also enhanced by their development into spheroids which secreted neurotrophic factors several times more than non-spheroid PNSCs and expressed several types of extra cellular matrix. These features suggest that the potential for these PNSC spheroids can overcome their limitations. In an animal spinal cord injury (SCI) model, these PNSC spheroids induced functional recovery and neuronal regeneration. These PNSC spheroids also reduced the neuropathic pain which accompanies SCI after remyelination. These PNSC spheroids may represent a new therapeutic approach for patients suffering from SCI.

## 1. Introduction

Spinal cord injury (SCI) is mainly caused by external injuries, but also by vascular disorders, tumors, and infections. SCI can cause medical complications that include temporary or permanent impairments of motor, sensory, and autonomic functions. Currently, only a few treatments exist for SCI: the administration of high doses of corticosteroids within one day after injury, decompression of the injured area by surgery, and pain relief [1,2,3,4]. There are no treatments for addressing the root causes or for restoring lost tissue, so gene and cell therapies, in addition to drug treatments, are the subjects of intense neuronal-regeneration research [5,6,7].

Stem cell therapy is a major field of research for SCI treatment. It has been reported to inhibit secondary damage by reducing inflammation and fostering axon and myelin regeneration. Preclinical and clinical studies have demonstrated effective stem cell therapies using bone marrow-derived mesenchymal stem cells (B-MSCs), cord-blood-derived MSCs, adipose-derived MSCs, neural stem cells (NSCs), induced pluripotent stem cells (iPSCs), embryonic-cell-derived oligodendrocyte progenitor cells (OPCs), and Schwann cells [2,8,9]. Among these, MSCs have been mostly used in SCI clinical trials. In these studies, the effects of MSCs were reported to be anti-inflammatory, immunomodulatory, anti-apoptotic, and paracrine, and included the release of neurotrophic factors. However, stem cells still have the potential risk for providing pro-tumorigenic influences, immune rejection due to pre-culture conditions, poor engraftment/differentiation/viability, and poor therapeutic patient outcomes [8,10,11,12,13]. The origins of NSCs used for SCI research are from several sources: fetal and embryonic stem cells, iPSCs, and via trans-differentiation [14]. NSCs also have been shown to have neuroprotective and anti-inflammatory effects, the ability to differentiate into neurons, and the ability to release neurotrophic factors. However, ethical and safety concerns remain about potential tumorigenesis due to their sources [8]. Due to these reasons, identification of other cell types for effective SCI therapies is needed, with the possibility of pre-conditioning them or modifying their genes to enhance both their survival and paracrine effect.

The neural crest stem cells (NCSCs) have been shown to migrate along nerve fibers for wide distribution, where they give rise to a variety of neural crest (NC)-lineage cells ranging from neurons and glia of the peripheral nervous system to non-neural cells, including melanocytes, endocrine cells, endoneurial fibroblasts, pericytes, chondrocytes, osteoblasts, and MSCs [15,16,17,18,19,20]. Even though these embryonic cells disappear quickly after birth, studies have reported the existence of NCSC-like cells in a variety of postnatal NC-derived tissues, including the dorsal root ganglion ganglion [21], gut [22], cornea [23], heart [24], dental pulp [25], skin [26], hair follicle [27], carotid body [28,29], palatum [30], and bone marrow [31]. These postnatal NSCS-like cells exhibit the ability to generate various types of NC-lineage cells. Moreover, their niches are intimately associated with PN [18,32].

Adult PN studies have reported endoneurial cells with the NCSC-like phenotypes expressing NC-related markers such as nestin, Pax3, and p75NTR [33,34], and a few studies have successfully isolated PNSCs from adult sciatic nerves [35,36]. These cells had the ability to differentiate into Schwann cells and into mesodermal-lineage cells and some were also able to form spheroids. However, the reported methods were technically demanding, time-consuming, and involved multiple steps, all of which resulted in low cell yields after long isolation and culture times. Considering their neurogliogenic abilities, postnatal PNSCs may represent a promising source for translational cell-based strategies applied to neurological disorders.

Typically, most stem cells are cultured using two-dimensional (2D) monolayer culture conditions and transplanted as single-cell suspensions. However, low biological efficacy and a low engraftment rates in hostile transplant environments remain concerns, preventing their use clinically. To overcome some of these limitations, three-dimensional (3D) culturing was developed. This approach was shown to be more effective as a therapeutic strategy due to enhanced biological efficacy, cell survival, and engraftment compared to conventional single-cell suspensions [37]. As an example of a typical 3D microstructure, multicellular spheroids consist of both cell-to-cell and cell-to-extracellular matrix interconnections, leading to enhanced structural stability and functional properties. Among many varieties of culturing conditions, suspension-culture conditions have become widely used to facilitate the formation of cell spheroids [38].

## 2. Results

### 2.1. 3D Organ Culture Supported Cell Renewal and Outgrowth of PNSCs

**Figure 1:**
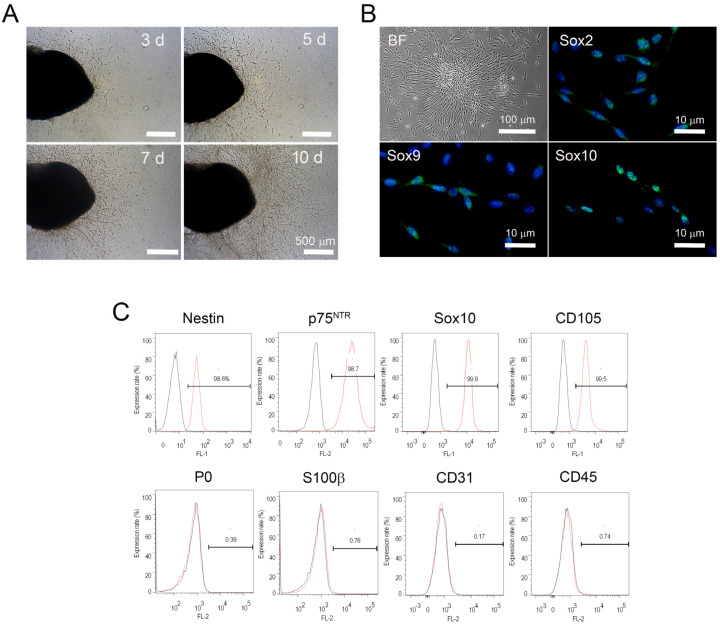
Isolation and immunophenotypic characteristics of peripheral nerve-derived stem cells (PNSCs). (**A**) Hydrogel-supported 3D organ culture of peripheral nerve (PN) supported cell migration/outgrowth. Cell migration/outgrowth increased in a time-dependent manner. (**B**) Migrated PNSCs in the hydrogels were isolated and then cultured under monolayer-culture conditions. PNSCs exhibited a bipolar spindle-shape appearance and expressed neural crest-specific transcription factors. (**C**) Flow cytometry assessment demonstrated that isolated PNSCs expressed markers for neural crest-lineage cells but did not express markers for myelin (P0), Schwann cells (S100β), or endothelial and hematopoietic cells (CD31 and CD45, respectively).

We adopted 3D hydrogel-supported organ culture to support in situ PNSC cell renewal and migration from PN into the hydrogel. As shown in Figure 1A, the neutral collagen hydrogel used here had a sol-to-gel transition property that allowed PN fragments to be 3D encapsulated in the gel phase, and it enabled tissue fragments to provide a 3D substratum for cell attachment, migration, and proliferation of PNSCs. During these hydrogel-supported PN organ cultures, PNSC outgrowth into the hydrogel was initially observed within one day of culture, after which the outgrowth showed a time-dependent increase. All hydrogel-supported PN cultures showed consistent cell outgrowth. After 14 days of culture, the migrated cells were isolated from the hydrogels by treatment with 0.01% collagenase type I, which led to the rapid degradation of collagen hydrogel but had no lytic effect on tissue. The migrated PNSCs that were released from the hydrogels were collected by centrifugation, suspended in cell-culture media, transferred to polystyrene culture dishes, and then subcultured using monolayer culture conditions as described in Methods and Materials.

We next assessed the immunophenotypic profiles of isolated and subcultured PNSCs. PNSCs uniformly expressed Sox2, Sox9, and Sox10; these transcription factors specifically identify NC-lineage cells (Figure 1B). We further investigated the immunophenotypic profiles of isolated PNSCs using flow cytometry. As shown in Figure 1C, assessed PNSCs also expressed NC-specific markers (nestin, p75NTR, Sox10, and CD105), but not markers for myelinating Schwann cells (P0 and S100b), endothelial cells (CD31), and hematopoietic cells (CD45).

### 2.2. Isolated PNSCs Showed Multipotent Differentiation Potential for Ectomesoderm-Lineage Cells In Vitro

**Figure 2:**
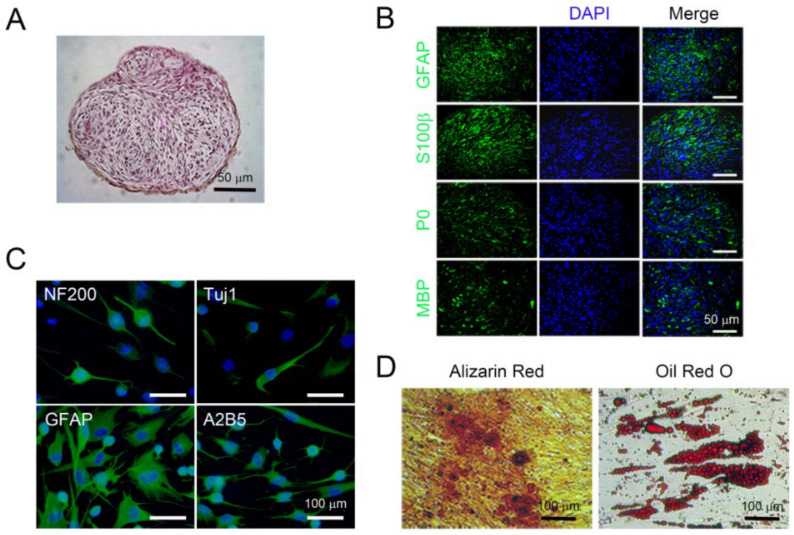
Multipotent differentiation potential of isolated peripheral nerve-derived stem cells (PNSCs) in vitro. (**A**) Isolated PNSCs were induced to become Schwann cell-like cells. Under suspension-culture conditions for seven days, PNSCs formed multicellular spheroid structures with fascicular arrangements. (**B**) Spheroid PNSCs expressed markers for committed Schwann cells (GFAP and S100β) and for myelin (P0 and MBP). (**C**) PNSCs induced to become neuroglia-lineage cells expressed neuronal markers (NF200 and Tuj1), a glial marker (GFAP), and an oligodendrocyte marker (A2B5). (**D**) PNSCs induced to become osteocytes and adipocytes exhibited depositions of mineral crystals and cytoplasmic accumulation of fat vacuoles visualized by Alizarin Red and Oil Red O staining, respectively.

To test whether PNSCs have the ability to differentiate into Schwann cells in vitro, cells were seeded onto Ultra-Low attachment plates and cultured for seven days without any differentiation-inducing factors. PNSCs assembled into spheroids and showed fascicular arrangements of spindle-shaped cells, mimicking PN bundles (Figure 2A). These spheroid PNSCs uniformly expressed Schwann cell-related markers (GFAP and S100β) as well as myelin-specific markers (P0 and MBP), indicating that PNSCs have an ability to differentiate into myelinating Schwann cells (Figure 2B). Next, we assessed whether isolated PNSCs were to differentiate into neuroglia-lineage cells. After 14 days of differentiation-induction, less than 10% of cells were neuron-like in appearance (extensive bipolar and branched neurites that expressed NF200 and Tuj1; Figure 2C). Moreover, a majority of cells (>80%) showed the typical bush-like morphology of protoplasmic astrocytes and expressed GFAP. A minority of cells (<10%) demonstrated oligodendrocyte-like morphology and expressed A2B5. A key feature of embryonic NC cells is the ability to differentiate into mesoderm-lineage cells, such as adipocytes and osteocytes. To determine if PNSCs also had this ability in vitro, PNSCs were induced to become adipocytes and osteocytes. These induced PNSCs exhibited cytoplasmic accumulations of multivacuolated fat vacuoles and mineral pigments, indicating that PNSCs have the ability in vitro to differentiate into adipocytes and osteoblasts, respectively (Figure 2D).

### 2.3. PNSC Spheroids Maintained the Immunophenotype of Schwann Cell-Lineage Cells and Promoted Pro-Neurotrophic Activity

**Figure 3:**
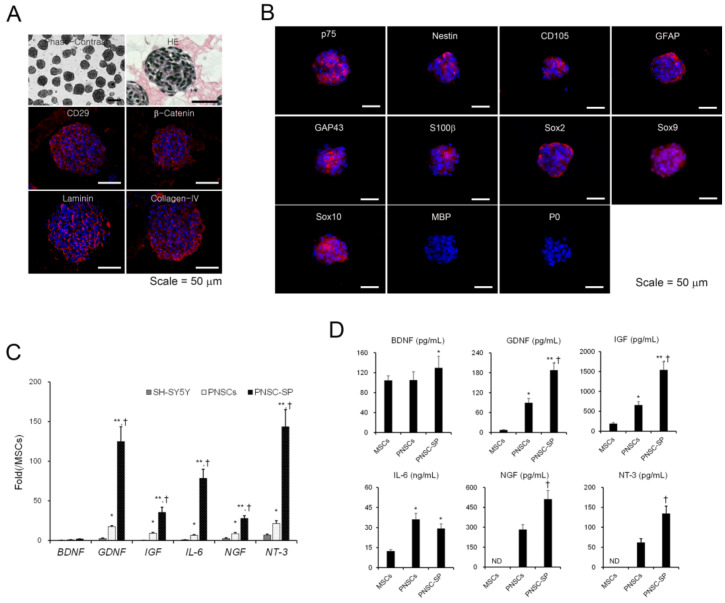
Structural, immunophenotypic, and biomolecular characteristics of peripheral nerve-derived stem cell spheroids (PNSCs). (**A**) Under suspension-culture conditions for three days, PNSCs spontaneously assembled into spheroid structures. (**B**) PNSC spheroids exhibited cell-to-cell and cell-to-matrix interconnections through integrin-β1 (CD29) and β-catenin, respectively. Notably, the in vitro deposition of synthesized extracellular matrix in spheroids was observed. (**C**,**D**) PNSCs and PNSC spheroids highly expressed and secreted neurotrophic factor mRNAs and proteins compared to mesenchymal stem cells (MSCs) derived from bone marrow. *, *p* < 0.05 compared to MSCs, **, *p* < 0.01 compared to MSCs, †, *p* < 0.01 compared to PNSCs.

Under suspension-culture conditions, PNSCs self-assembled or aggregated to form 3D spheroids (Figure 3A). These spheroids exhibited tight adhesions between cells with eukaryotic nuclei, and necrotic/apoptotic cells were not observed during three days of culture. Immunofluorescent staining demonstrated cell-to-cell and cell-to-matrix adhesions mediated by integrin-β1 (CD29) and β-catenin. Interestingly, the uniform deposition of synthesized extra cellular matrix (ECM) in vitro (e.g., laminin and collagen type-IV) during suspension culture was also detected in PNSC spheroids. These interactions between cells and ECM may not only have led to increased structural integrity but may also have activated cell-signaling pathways to regulate cell differentiation.

To determine the immunophenotypes of PNSC spheroids after three days of suspension culture, immunofluorescence staining was performed with antibodies to identify NC- and Schwann cell-lineage cells. PNSC spheroids uniformly maintained their expressions of NC-lineage markers, including nestin, p75NTR, and CD105 (Figure 2B). Moreover, NC-specific transcription factors (e.g., Sox2, Sox9, and Sox10) were also maintained in PNSC spheroids. Notably, markers representing further commitment to the Schwann-cell lineage (e.g., GFAP, GAP43, and S100b) were also expressed in PNSC spheroids, whereas these markers were not expressed in earlier PNSC experiments. However, markers for the myelin sheath (MBP and P0) were not detected in PNSC spheroids. These immunophenotypic features suggest that PNSCs assembled in spheroids showed preferential differentiation into the Schwann-cell lineage most similar to non-myelinating or dedifferentiated Schwann cells.

Accumulating evidence suggests that 3D spheroids can significantly modulate a variety of cell-signaling pathways to upregulate the expression of many functional genes and enhance their therapeutic potential. To compare neurotrophic activities between PNSCs and PNSC spheroids, we assessed the expression levels of neurotrophic factor mRNAs and their secretion levels by qRT-PCR and ELISA, respectively. PNSCs expressed high levels of neurotrophic factor mRNAs compared to their levels in MSCs. As expected, 3D spheroids significantly upregulated the expression levels of the neurotrophic factors compared to PNSCs (*p* < 0.01) and MSCs (*p* < 0.01; Figure 3C). After collecting conditioned media from PNSCs, PNSC spheroids, and MSCs, we determined the secretion levels for the neurotrophic factors. Interestingly, PNSCs secreted high levels of glia-related neurotrophic factors, including cell-line-derived neurotrophic factor (GDNF), insulin-like growth factor (IGF), and interleukin (IL)-6, compared to levels in MSCs (*p* < 0.01; Figure 3D). Both nerve growth factor (NGF) and neurotrophin-3 (NT-3) were detected in conditioned media from PNSCs but not detected in MSCs. Moreover, the secretion levels of GDNF, IGF, NGF, and NT-3 were significantly increased in PNSC spheroids compared to PNSCs (*p* < 0.01). Collectively, PNSCs showed specific high expression and secretion of glia-related neurotrophic mRNAs and factors, and the neurotrophic activity of PNSCs can be potentiated through the formation of 3D spheroids.

### 2.4. PNSC Spheroids Improved Functional Recovery after SCI

**Figure 4:**
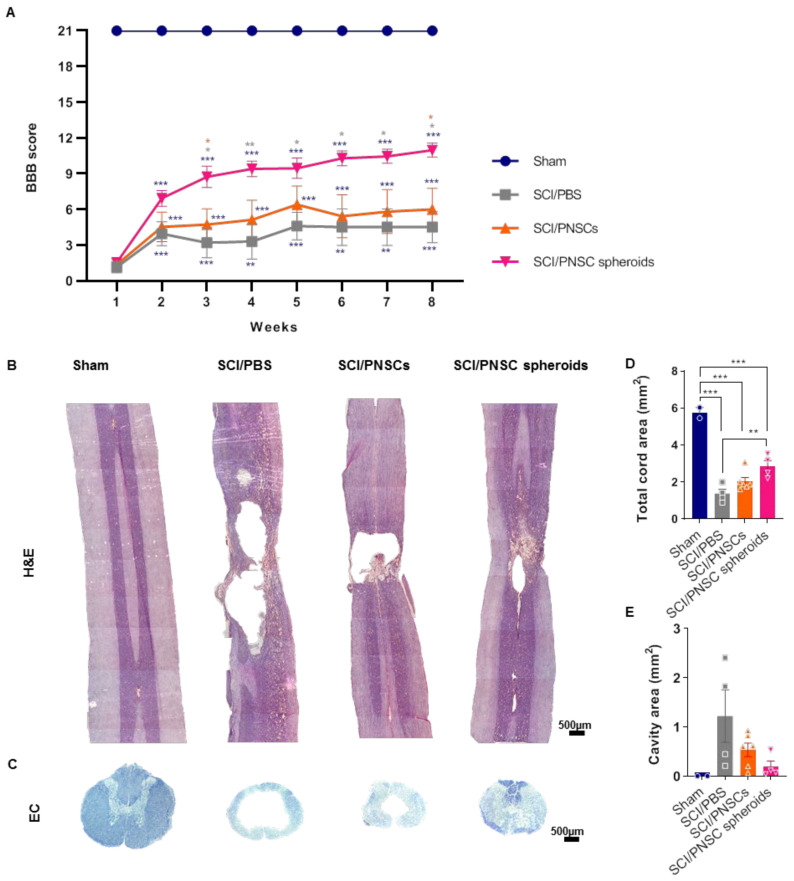
Peripheral nerve-derived stem cell (PNSC)-mediated functional recovery and neuronal regeneration. (**A**) BBB scoring after SCI in all groups (Sham, SCI/PBS, SCI/PNSCs, and SCI/PNSC spheroids). A two-way ANOVA with Holm–Šídák’s multiple comparisons was used for data analysis. (**B**) Hematoxylin and eosin staining of longitudinal spinal cord sections in all groups for assessment of tissue regeneration. (**C**) Eriochrome cyanine (EC) staining of spinal cord cross sections for myelin in all groups. (**D**) Total cord area remaining using EC imaging in Sham (*n* = 3), SCI/PBS (*n* = 4), SCI/PNSCs (*n* = 6), and SCI/PNSC spheroids (*n* = 4) animals. Ordinary one-way ANOVA with Tukey’s multiple comparisons was used for analysis. (**E**) Total cavity area from EC imaging. Ordinary one-way ANOVA with Tukey’s multiple comparisons was used for analysis. All data are expressed as mean ± S.E.M.; * *p* < 0.05, ** *p* < 0.01, and *** *p* < 0.001.

To investigate the therapeutic effects of PNSCs, we used a compression-based SCI model in rodents. PNSCs were directly injected either as single-cell suspensions or as spheroids one week after injury. Open-field locomotor testing was performed weekly for eight weeks and Basso, Beattie, and Bresnahan (BBB) scores were used to evaluate locomotion [39]. After SCI, the SCI/phosphate-buffered saline (PBS) group showed significantly decreased motor function. The SCI/PNSCs group (non-spheroid PNSCs) showed a slight behavioral improvement, but it was not a significant change (Figure 4A). In contrast, BBB scores gradually and significantly increased in the SCI/PNSC spheroids group (Figure 4A). Eight weeks after injury, the mean SCI/PNSC spheroids BBB score was 10.175 ± 0.95, indicating weighted walking using the hindlimbs. These results indicate that PNSCs have therapeutic value, and that the spheroid form enhances it.

To confirm correlations between functional recovery and tissue regeneration, the spinal cords from each group were stained using hematoxylin/eosin and eriochrome cyanine (EC) (Figure 4B,C). In the sham group, there was no evidence of spinal cord degeneration, but large cavities and tissue shrinkage were observed in the SCI/PBS group. In the SCI/PNSCs group spinal cords, there was also tissue shrinkage and the cavities were reduced, but still severe. In contrast, spinal cord tissue was regenerated close to sham levels in the SCI/PNSC spheroids group (Figure 4B). In addition, the entire spinal cord area was restored, and the cavity areas were significantly decreased in the SCI/PNSC spheroids group (Figure 4D,E). As these results correlated well with the BBB scores it suggests that PNSCs induce spinal cord regeneration, especially in the SCI/PNSC spheroids group, and are responsible for the functional recovery.

### 2.5. PNSC Spheroids Reduced Mechanical Allodynia and Inflammation Following SCI

**Figure 5:**
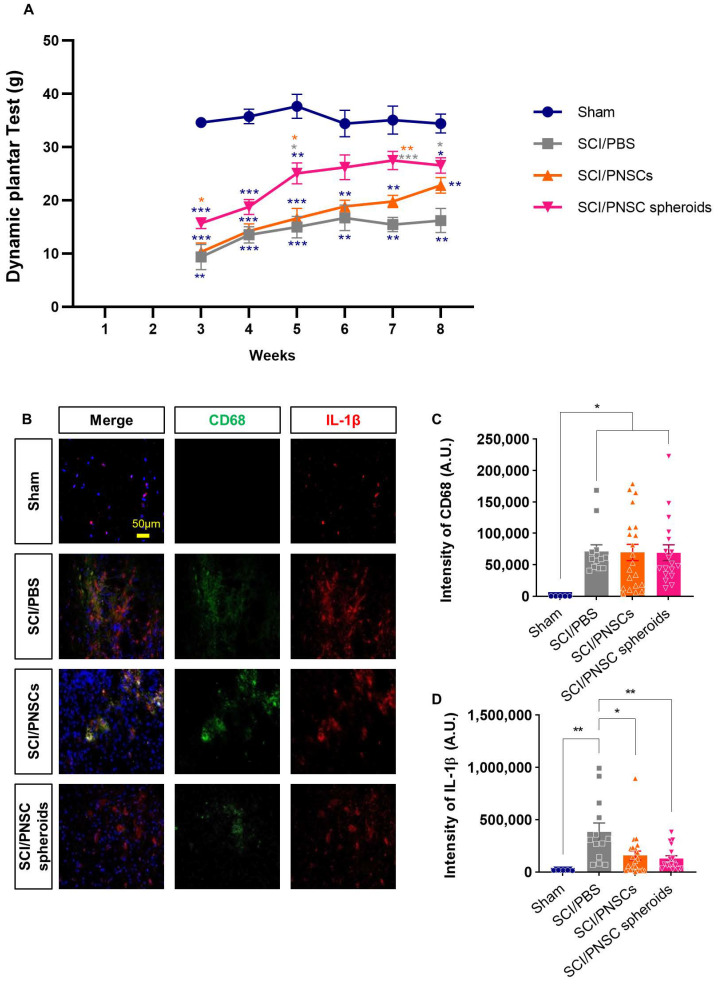
Peripheral nerve-derived stem cell (PNSC)-mediated improvement in mechanical allodynia and inflammation. (**A**) Mechanical allodynia assessment using a dynamic plantar test. Testing began three weeks after SCI and continued until eight weeks after SCI. Data were analyzed by using a two-way ANOVA with Holm–Šídák’s multiple comparisons. (**B**) Representative imaging showing DAPI (blue), anti-CD68 (green), and anti-IL-1β (red) staining eight weeks after SCI. (**C**) Total CD68 staining intensity in all groups (Sham, *n* = 5; SCI/PBS, *n* = 13; SCI/PNSCs, *n* = 21; and SCI/PNSC spheroids, *n* = 28). Ordinary one-way ANOVA with Tukey’s multiple comparisons was used for analysis. (**D**) Total IL-1β staining intensity in all groups (Sham, *n* = 5; SCI/PBS, *n* = 13; SCI/PNSCs, *n* = 21; and SCI/PNSC spheroids, *n* = 28). Ordinary one-way ANOVA with Tukey’s multiple comparisons was used for analysis. All data are expressed as mean ± S.E.M.; * *p* < 0.05, ** *p* < 0.01, and *** *p* < 0.001.

Previous reports have demonstrated that neuropathic pain can accompany SCI [40]. Here, we confirmed mechanical allodynia using a dynamic plantar test [41]. Mechanical allodynia was measured beginning three weeks after the SCI when the animal hind paws were facing down. The sham-operated group withdrew their hind paws using an average force of 31 g. Three weeks after injury, animals in the SCI/PBS and SCI/PNSCs groups were hypersensitive to mechanical stimulation. Eight weeks after injury, they were still hypersensitive, removing their hind paws at 16.2 and 22.8 g, respectively. In contrast, the SCI/PNSC spheroids group withdrew their paws at 15 g three weeks after injury, indicating less sensitivity to stimulation compared to the SCI/PBS and SCI/PNSC groups. Overall, the SCI/PNSC spheroids group showed mechanical allodynia improvement that was close to sham eight weeks after SCI (Figure 5A).

Mechanical allodynia is only one form of neuropathic pain and the causes of neuropathic pain after SCI are not known, but those suspected include immune responses, glial cells, and myelin degeneration [40,42,43,44]. Here, we confirmed an inflammatory response using the markers CD68 and IL-1β (Figure 5B). In all SCI animals, CD68 expression was significantly increased, with no significant differences between the SCI/PBS, SCI/PNSCs, and the SCI/PNSC spheroid groups (Figure 5B,C). However, IL-1β expression (a pro-inflammatory cytokine implicated in neuropathic pain [45]) differed significantly between groups. IL-1β expression was reduced to 33% in the SCI/PNSC spheroids group compared to the SCI/PBS group (Figure 5B,D). This result also correlates with the mechanical allodynia results and indicates that PNSC spheroids improve neuropathic pain.

### 2.6. PNSCs Survived Three Weeks after Transplantation and Affected Both Neurons and Glial Cells

**Figure 6:**
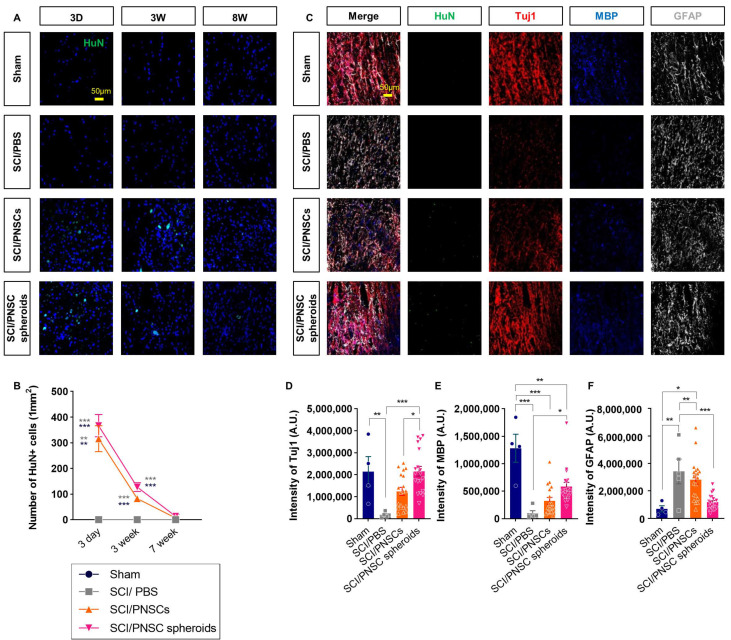
Viability and differentiation of transplanted peripheral nerve-derived stem cells (PNSCs). (**A**) Representative confocal imaging showing DAPI (blue) and anti-HuN (green) staining three days, three weeks, and seven weeks after transplantation. (**B**) Total number of HuN+ with DAPI+ cells per mm^2^. (**C**) Representative confocal imaging showing anti-HuN (green), anti-Tuj1 (red), anti-MBP (blue), and anti-GFAP (white) eight weeks after injury. (**D**) Total Tuj1 staining intensity in the Sham (*n* = 4), SCI/PBS (*n* = 5), SCI/PNSCs (*n* = 19), and SCI/PNSC spheroids (*n* = 19) groups. Ordinary one-way ANOVA with Tukey’s multiple comparisons was used for analysis. (**E**) Total MBP staining intensity in all groups. The *n* numbers and analysis was the same as for Tuj1. (**F**) Total GFAP staining intensity in all groups. The *n* sizes and analysis were the same as for Tuj1. All data are expressed as mean ± S.E.M.; * *p* < 0.05, ** *p* < 0.01, and *** *p* < 0.001.

We assessed cell survival three days, three weeks, and seven weeks after cell transplantation. Transplanted PNSCs were identified using HuN because they were originally isolated from adult humans. Three days after transplantation, almost all cells survived, but only 27% and 31% of cells survived three weeks after transplantation (compared to the three-day numbers) in the SCI/PNSCs and SCI/PNSC spheroid groups, respectively (Figure 6A,B). Seven weeks after transplantation, almost no HuN-positive cells were observed in all groups (Figure 6A,B). These results indicate that single-cell suspensions and PNSC spheroids do not survive long term, but they still affected the spinal cord environment to improve functional behavior.

The PNSCs used in this study have the potential to differentiate into both neurons and neuroglial cells. We therefore investigated the neurons, astrocytes, and oligodendrocytes with antibodies against Tuj1, GFAP, and MBP eight weeks after injury (Figure 6C). Differentiated PNSCs were not observed because the cells did not survive seven weeks after transplantation. Although differentiated cells were not observed, the expressions of these neuronal and glial cell markers changed significantly. In the PNSC-transplanted groups, the expressions of the neuronal marker Tuj1 and the oligodendrocyte marker MBP increased, but expression of the astrocytic marker GFAP decreased (Figure 6C–F). This was especially true for the SCI/PNSC spheroids group which showed the most marked changes in all cell types; Tuj1 expression increased significantly to near-sham levels and the expressions of the other markers was closer to sham levels compared to the other groups (Figure 6C–F). These results indicate that PNSC viability is not sufficient; its paracrine effects are required for the enhancement of recovery and is improved by the formation of spheroids.

### 2.7. Transplanted PNSCs Induced the Expression of Neurotrophic Factors

**Figure 7:**
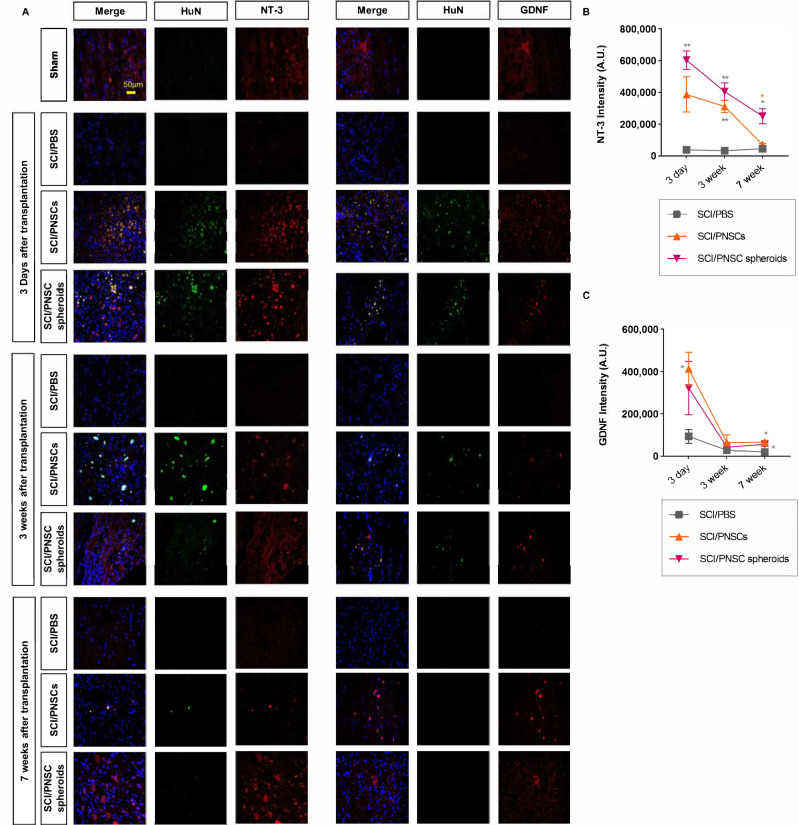
The expressions of GDNF and NT-3 after SCI. (**A**) Representative confocal imaging, with blue indicating DAPI, green indicating HuN, and red indicating either NT-3 or GDNF. (**B**) Total NT-3 staining intensity, with *n* = 5 for all groups. Data were analyzed using a two-way ANOVA with Holm–Šídák’s multiple comparisons. (**C**) Total GDNF staining intensity, with *n* = 5 for all groups. Data were analyzed using a two-way ANOVA with Holm–Šídák’s multiple comparisons. All data are expressed as mean ± S.E.M.; * *p* < 0.05, ** *p* < 0.01.

From the above results, the paracrine influence of transplanted PNSCs was expected to be via neurotrophic factor expression (Figure 3C,D and Figure 6C). This was especially true for the increases in GDNF and NT-3 expressions in PNSC spheroids (Figure 3C,D). Neurotrophic factors play important roles in both neuroprotection and neuronal regeneration. Three days after transplantation, NT-3 expression was significantly increased in both the SCI/PNSC and the SCI/PNSC spheroid groups. Three weeks after transplantation, NT-3 expression was also increased in both of these PNSC groups. Even though increased NT-3 expression was not maintained in the SCI/PNSC group, it remained so in the SCI/PNSC spheroids group (Figure 7A,C).

The other neurotrophic factor examined, GDNF, was also increased in the SCI/PNSC and SCI/PNSC spheroid groups three days after transplantation similar to NT-3 (Figure 7B,D). However, this increased GDNF expression was not maintained seven weeks after transplantation, with levels close to those of the SCI/PBS group beginning three weeks after transplantation in the SCI/PNSC and SCI/PNSC spheroid groups (Figure 7B,D).

### 2.8. PNSC Spheroids Enhanced Remyelination after SCI

Recovery after a SCI is related not only to neuronal regeneration but also to remyelination [46,47]. Here, we demonstrated PNSC spheroid myelination ability using EC staining (Figure 4C) and using an antibody against MBP (Figure 6C). Seven weeks after transplantation, PNSC single-cell suspensions induced MBP expression (Figure 6C,E) and the entire area was largely myelinated compared to the PBS transplanted group, but this change was not statistically significant (Figure 4C). In contrast, PNSC spheroids significantly enhanced MBP expression (Figure 6C,E) and myelination in the area was significantly increased so that even gray and white matter could be distinguished from each other (Figure 4C). These results indicate that PNSC spheroids can improve remyelination after SCI.

## 3. Discussion

Here, we report on the development of PNSC spheroids that: (1) can form as stable spheres (100 ± 20 μm); (2) have immune expression characteristics similar to NC stem cells; (3) have the ability to differentiate into neuronal and mesenchymal cells; (4) consistently release several neurotrophic factors and anti-inflammatory factors; and (5) have improved therapeutic ability in a SCI model.

Stem cell therapy represents one of the strategies for SCI treatment. Several stem cell types have been used for SCI treatments, including B-MSCs, cord-blood-derived MSCs, and NSCs. Because these stem cells have the potential for multilineage differentiation, their transplantations were expected to engraft and replace damaged cells [2,8]. However, the conditions within the injured environment make the survival of transplanted cells difficult and lower the probability for both engraftment and differentiation [8,10,11,12,13]. For these reasons, genetic modifications and preconditioning in vitro have been attempted to increase any paracrine effects and to increase cell survival [48,49]. Stem cells have been reported to inhibit secondary damage, have anti-inflammatory effects, protect host cells, and produce neurotrophic factors [2,8,50]. The present report documents our development of adult PNSCs and PNSC spheroids. Unlike the central nervous system, PNs have the capacity for self-repair and regeneration. After a PN injury, the repair-type Schwann cell is generated through the de-differentiation or reprogramming pathway. It plays a central role in PN regeneration by releasing both neuroactive and anti-inflammatory factors [51]. These repair-type Schwann cells also share similar features with NCSCs and Schwann-cell precursors [51,52,53]. The adult PNSCs have similar immunophenotypes to undifferentiated NCSCs. These PNSCs express neurotrophic factor mRNAs and proteins for BDNF, GDNF, IGF-1, IL-6, NGF and NT-3. As mentioned above, stem cells have been modified genetically and via preconditioning to improve therapy effectiveness. Here, we developed PNSC spheroids with increased viability, which released several-fold increases in neurotrophic factors and anti-inflammatory factors compared to non-spheroid PNSCs, and which generated extracellular matrix components including fibronectin, laminin, and collagen type-IV.

Because of these features, the long-term survival of these PNSC spheroids was three weeks after transplantation. Although PNSCs did not survive seven weeks after transplantation, regeneration was enhanced after spinal cord injury and they induced functional recovery. In the SCI/PNSC spheroids group, the neuronal marker Tuj1 was significantly increased, the astrocytic marker GFAP was significantly decreased and the final expression levels for both markers approached those of the sham group. Although these PNSCs demonstrated substantial differentiation potential and the possibility for engraftment via ECM expression, the survival of transplanted PNSCs was not enough for engraftment and differentiation. The effects of these PNSCs were based on paracrine influences of neurotrophic factor expression. From the SCI/PNSC spheroids group, both GDNF and NT-3 were highly and continuously expressed. These two factors are known to have both neuroprotective effects and to induce neuronal regeneration [54,55,56]. Other changes, such as the significant PNSC-mediated decrease in astrocytes, may have mixed outcomes after SCI because reactive astrocytes have been shown to protect host cells from secondary damage in acute-phase SCI, but can interrupt neuronal regeneration in chronic-phase SCI. One of the increased neurotrophic factors, GDNF, is also known as a powerful protector of neuronal cells, and is mostly expressed in glial cells [54,55,56,57]. GDNF has also been reported to reduce GFAP expression and hypertrophy of reactive astrocytes [58]. It is likely that GDNF reduced reactive astrocytes especially in the SCI/PNSC spheroids group and that GDNF expression was relatively lower in the SCI/PNSC spheroids group. These differences are also likely to account for the SCI/PNSC spheroids group showing the most impressive functional recovery and regeneration. The other neurotrophic factor, NT-3, has also been shown to prevent cell death, reduce atrophy, and to induce neuronal regeneration [59]. In the SCI/PNSC spheroids group, NT-3 was significantly increased compared to the other SCI groups, and its increased expression was maintained until seven weeks after transplantation even though PNSCs no longer survived. All of these results can be explained by PNSC paracrine effects; transplanted PNSCs and PNSC spheroids influenced host cells and maintained that influence until seven weeks after transplantation. This can also explain the increased GDNF expressed in astrocytes, and its rare expression in the SCI/PBS group. In addition, most NT-3 and GDNF expression was seen in host cells seven weeks after transplantations.

We have demonstrated not only PNSC-mediated functional recovery but also mitigation of neuropathic pain. Neuropathic pain is a commonly reported medical complication in SCI patients [60,61,62]. Approximately 80% of patients suffer with pain during the acute phase of SCI, and 30–50% of patients suffer from it chronically, so the use of pain-relief medication is common in SCI patients [61]. Neuropathic pain due to SCI has several causes: inflammation, changes in intracellular signaling, reactive oxygen species, and demyelination [63,64]. Among these causes, SCI-induced demyelination can sometimes lead to secondary damage and neuropathic pain due to hyperexcitability [64,65]. Such demyelination can affect C-fibers by inducing the spontaneous stimulations that result in both hyperalgesia and allodynia [66,67]. Due to this, remyelination in the injured spinal cord is an important factor mediating functional recovery. Transplanted PNSC spheroids had a strong remyelination effect in the present SCI model, likely mediated by their high NT-3 expression. Previous reports have demonstrated that NT-3 increased mature oligodendrocytes and induced remyelination [68,69]. As an additional cause of neuropathic pain, inflammation was also significantly reduced in the SCI/PNSC spheroids group. Overall, the complex actions of PNSC spheroids improved both motor function and neuropathic pain after SCI.

The MSCs derived from bone marrow (B-MSCs) have been widely used as a cell therapeutic for the repair of SCI. Transplanted MSCs mediate their beneficial effect by secreting anti-inflammatory and anti-apoptotic factors that mitigate a secondary inflammatory response and cell death, respectively. However, the low secretion level of neurotrophic factors was one of limitations for their therapeutic potential in functional and structural recovery of injured spinal cord. To overcome these limitations, fetal neural stem cells (NSCs) have been tried based on their ability to secrete neurotrophic factors, which mediate their neuroprotective effects. However, clinical translation of these cells was hindered by the feasibility of obtaining sufficient quantities of tissue for isolating NSCs. In this manuscript, we first demonstrate that PNSCs express and secrete high levels of glia cell-derived neurotrophic mRNAs and factors that mediate their indirect neuroprotective effects. Moreover, in practical situations, obtaining adult peripheral nerves for isolating stem cells is easier than fetal tissue. Overall, the neurotrophic ability of PNSC might be used as a promising cell source for the treatment of SCI.

In conclusion, we have isolated adult stem cells from PNs with similar characteristics to undifferentiated NCSCs. For a more effective therapeutic approach to SCI, we developed PNSC spheroids using 3D culture. These PNSC spheroids showed a powerful therapeutic effect in a SCI model compared to single-cell PNSC suspensions. However, both cell differentiation and engraftment were not observed. Despite these limitations, these novel spheroids may represent a new clinical treatment for managing SCI.

## 4. Materials and Methods

### 4.1. Reagents

The PNs used for cell isolation were obtained from common iliac nerve segments harvested after brain death from human organ donors. This study was reviewed and approved by the Institutional Review Board of Inje University Busan Paik Hospital. Porcine skin-derived collagen (Matrixen^®^-PSC) and collagenase type I were obtained from SK Bioland (Chunan, Korea) and Worthington Biochemical (Lakewood, NJ, USA), respectively. Growth factors, including recombinant epidermal growth factor (EGF), basic fibroblast growth factor (bFGF), β-nerve growth factor (NGF), brain-derived neurotrophic factor (BDNF), and transforming growth factor-β1 (TGF-β1), were purchased from Peprotech (Seoul, Korea). Culture media, animal sera, and culture reagents were obtained from Invitrogen (Seoul, Korea), unless otherwise specified.

### 4.2. 3D Organ Culture and Cell Isolation

After removing the epineurium and surrounding connective tissue using a stereomicroscope, PN segments were minced using a razor blade into 2- to 3-mm-long pieces, washed with phosphate-buffered saline (PBS), and then suspended in a pre-chilled 0.25% neutral collagen solution. A mixture of 100 mg nerve pieces suspended in 10 mL of neutral collagen solution was transferred into a 100 mm tissue culture dish and then incubated in a humidified chamber for 2 hr at 37 °C to establish a neutral collagen hydrogel that would enable 3D encapsulation of the PN pieces. Fifteen milliliters of organ culture medium (100 ng/mL EGF, 20 ng/mL bFGF, and 10 μg/mL gentamicin in Dulbecco’s Modified Eagle Medium/Nutrient Mixture F-12 (DMEM/F12)) was added. Organ cultures were performed under dynamic conditions in an orbital shaker at 25 rpm for two weeks. The organ culture medium was replenished three times per week.

After 14 days of organ culture, collagen hydrogels containing PN fragments and outgrown PNSCs were incubated with 0.01% collagenase type I at 37 °C under dynamic conditions in an orbital shaker at 25 rpm for 30 min. When the hydrogels had degraded, the migrated/outgrown PNSCs released from the hydrogels were collected, centrifuged at 150× *g* for 10 min, and then suspended in cell culture medium (DMEM/F12 supplemented with 10 ng/mL EGF, 2 ng/mL bFGF, and 10% fetal bovine serum). These isolated PNSCs were seeded in polystyrene (PS) culture dishes and expanded using conventional monolayer-culture conditions. When the cells reached 80% confluence, PNSCs were detached using trypsin-EDTA treatment, seeded at a density of 5000 cells per cm^2^, and then subcultured at a 1:6 split ratio. This initial subculture under monolayer-culture conditions was considered passage 1 (P1). Subcultured PNSCs were used at passage 6 (P6) to determine transcriptional, immunophenotypic, and biologic properties, and at P5 for cell transplantation experiments, unless otherwise specified. All assays were carried out using three different batches of PNSCs and were performed at least twice for validation, unless otherwise specified. MSCs were derived from bone marrow using a conventional method, as described elsewhere [70], and used as negative controls. SH-SY5Y cells were obtained from ATCC (Manassas, VA, USA) and used as positive controls.

### 4.3. 3D Spheroid Cultures

PNSCs expanded in vitro were collected after trypsin-EDTA treatment and resuspended in suspension-culture medium composed of DMEM/F12 supplemented with 0.1% calf serum. PNSCs (7.5 × 106) were seeded in T75 Ultra-Low attachment cell culture flasks (Corning Inc., Corning, NY, USA). After 72 h of culture, established PNSC spheroids were collected from plates, washed three times with PBS, and then suspended in 1 mL of PBS (7.5 × 106 PNSCs) for transplantation.

### 4.4. Immunophenotype Analysis

The immunophenotypes of isolated and subcultured PNSCs were assessed by immunostaining using a set of antibodies against markers related to NC-lineage cells, Schwann cells, neuroglia, endothelial cells, and hematopoietic cells (for supporting information, see Table S1). The expression of a given marker was measured either by flow cytometry (FACS CantoTM, BD Bioscience, San Jose, CA, USA) or using confocal microscopy (Zeiss LSM 510 Meta, Gottingen, Germany). For immunofluorescence assessment, 3 × 104 cells were seeded in 8-well chamber slides (Lab-Teck™ Chamber Slide System, Thermo Fisher Scientific, Wilmington, DE, USA), cultured for one day, and then fixed using a 1:1 mixture of acetone and methanol. After incubation with 5% bovine serum albumin (Fraction V, IgG-free; Sigma Aldrich, Saint Louis, MO, USA) for 30 min, the cells were incubated with primary antibodies at 37 °C for 2 h. Isotype-matched secondary antibodies conjugated with Alexa Fluors (Invitrogen) were applied, and the cells were incubated at 37 °C for 30 min. Cell nuclei were counterstained with 2 μg/mL diamidino phenylindole (DAPI; Invitrogen) and coverslips were applied using ProLong^®^ Gold antifade reagent (Invitrogen). Immunophenotyping of PNSCs was further validated using flow cytometry by incubating 2 × 105 PNSCs with primary antibodies, and then labeling them with Alexa Fluor-conjugated anti-mouse, anti-rabbit, or anti-goat IgGs. A minimum of 10,000 events and 3000 cells were assessed to determine expression by flow cytometry or confocal microscopy, respectively. To assess the immunophenotypic characteristics of PNSC spheroids, they were washed with PBS, fixed with 3% paraformaldehyde for 10 min, rinsed with PBS, and then permeabilized with 0.2% Triton X-100 PBS for 2 h. Spheroids were incubated for 1 h with primary antibodies, washed three times with PBS, and then incubated with the appropriate Alexa Fluor-conjugated secondary antibodies. Nuclei were stained with DAPI and images were obtained using a scanning confocal microscope.

### 4.5. In Vitro Differentiation Potential

To test the differentiation potential of PNSCs to become Schwann cells in vitro, we used suspension-culture conditions. Briefly, 5 × 104 PNSCs were seeded in 12-multiwell Ultra-Low attachment plates (Costar^®^, Sigma Aldrich) and the medium to induce Schwann cell differentiation was added. After seven days using suspension-culture conditions, the degree of Schwann cell differentiation was assessed by immunofluorescence staining, as described above, using Schwann cell-related markers including GFAP, S100β, P0, and MBP.

The differentiation ability of PNSCs to become neuroglia-lineage cells was tested by seeding 5 × 104 PNSCs onto fibronectin-coated coverslips in 24-well plates and adding 500 μL of neuroglia differentiation medium (DMEM/F12 supplemented with 50 ng/mL NGF, 50 ng/mL BDNF, and 1 mM cyclic adenosine monophosphate (cAMP, Sigma Aldrich)). After 14 days of culture, neuroglial differentiation was estimated by immunofluorescence staining using antibodies against neuronal markers (NF200 and Tuj1) and those for glia (GFAP and A2B5).

The adipogenic differentiation medium for PNSCs was composed of 90% DMEM, 10% CS, 0.5 mM 3-isobutyl-1-methylxanthine (Sigma Aldrich), 1 μM dexamethasone, 0.2 unit/mL insulin (Sigma Aldrich), and 200 μM indomethacin (Sigma Aldrich). The osteogenic differentiation medium was composed of 90% Minimum Essential Medium-α supplemented with 10% CS, 0.1 μM dexamethasone, 10 mM β-glycerol phosphate (Sigma Aldrich), and 50 μM ascorbic acid. These differentiation media were changed twice per week. Adipogenic or osteogenic lineage differentiation was assessed either by Oil Red O (Sigma Aldrich) or Alizarin Red S (Sigma Aldrich) staining for detecting cytoplasmic accumulations of lipid droplets or the extracellular deposition of calcium phosphate, respectively.

### 4.6. mRNA Expression

Transcriptional phenotyping of isolated PNSCs was determined using quantitative reverse transcription (RT)-polymerase chain reaction (PCR). Total RNA was obtained from PNSCs, MSCs, and SH-SY5Y cells using TRI-reagent^®^ (Molecular Research Center Inc., Cincinnati, OH, USA), according to the manufacturer’s instructions. The quantity of RNA was determined using a spectrophotometer (NanoDrop^®^ ND-1000, Thermo Fisher Scientific, Wilmington, DE, USA). One microgram of RNA was reverse-transcribed using random primers and SuperScript^®^ VILOTM (Invitrogen) to generate first-strand cDNA. This reaction was carried out in a GeneAmp 2400 PCR thermocycler (Applied Biosystems, Carlsbad, CA, USA) for 10 min at 25 °C, 50 min at 42 °C, and 15 min at 70 °C, followed by cooling to 4 °C. All PCR experiments were performed using an ABI Prism 7700 system (Applied Biosystems, Carlsbad, CA, USA). All the amplifications were done using a SYBR Green PCR Master Mix (Applied Biosystems). The thermal cycling conditions included an initial denaturation step at 95 °C for 10 min, followed by 40 cycles at 95 °C for 30 s, 60 °C for 30 s, and 72 °C for 30 s. PCR melting-curve analyses were conducted after each cycle. All samples were assayed in triplicate. For each sample, the expression of each gene was normalized to the expression of a housekeeping gene (GAPDH mRNA) using the 2-ΔΔCt method, where ΔCt = Ct, reference gene—Ct, target gene. Dissociation curves indicated that single products were obtained for each transcript. Information about the specific primers used for the neurotrophic factor mRNA sequences is listed in Table S2.

### 4.7. Cytokine Assay

Conditioned media samples (CM) from monolayer and 3D spheroid cultures (DMEM/F12 supplemented with 0.1% calf serum) were collected after 72 h and concentrated using Amicon^®^ Ultra-15 centrifugal filter units (Millipore, Billerica, MA, USA). Neurotrophic factor concentrations were measured using commercially available ELISA kits (R&D Systems, Minneapolis, MN, USA), including BDNF (DY248), GDNF (DY212), IGF (DY291), IL-6 (DY206), NGF (DY-256), and NT-3 (DY-267), according to the manufacturer’s instructions.

### 4.8. Spinal Cord Injury and Transplantation

In this study, SD rats (200 ± 20 g; OrientBio, Kyungki-do, Korea) were used and kept in an animal facility permitted by the Association for Assessment and Accreditation of Laboratory Animal Care (AAALAC). All animals were anesthetized with ketamine (100 mg/kg; Yuhan, Seoul, Korea), rompun (10 mg/kg; Bayer Korea, Seoul, Korea), and isotropy 100 (Troikaa Pharmaceuticals Ltd., Gujarat, India). Spinal cords were exposed by laminectomy in all animals. The exposed spinal cords of the SCI animals were compressed at the T9 level for 10 s using self-closing forceps (Fine Science Tools, North Vancouver, BC, Canada) [71]. Sham-group animals only had laminectomies without SCI. After surgery, the SCI animals were randomly divided into three groups: SCI/PBS, SCI/PNSCs (transplanted as single-cell suspensions), and SCI/PNSC spheroids (transplanted as spheroids). One week after injury, transplants of either PBS, PNSCs, or PNSC spheroids (5 × 105 cells/5 μL volume) were accomplished using a 27G Hamilton syringe. Injection rates were maintained as 0.5 μL/min and the needle was kept in place for an additional 5 min after each injection. After all surgical procedures, cefazolin (25 mg/kg; Chong Kun Dang, Seoul, Korea) was administered for 5 days and cyclosporine (20 mg/kg; Chong Kun Dang) was administered until the animals were sacrificed.

### 4.9. Open Field Testing

To assess functional recovery, open-field locomotor testing was performed weekly for eight weeks after injury. BBB motor scores [39] were determined to quantify animal movements. BBB scoring includes three-joint movement-angle ranges, weighted stepping, gait coordination, and tail movement, and has a range of 0 to 21. A score of 0 indicates complete paralysis and a score of 21 indicates normal movement. BBB scoring was performed by two different investigators blinded to animal grouping.

### 4.10. Mechanical Allodynia Measurement

Mechanical allodynia measurements were performed beginning three weeks after injury when the animal hind paws were positioned facing down. For these measurements, animals were placed in an acrylic enclosure on a wire-net platform for 10 min prior to mechanical stimulation. Stimuli were applied to plantar hind paws using a Dynamic Plantar Aesthesiometer (Ugo Basile, Comerio, Varese, Italy). A 0.5 mm diameter steel rod was pushed into hind paws with gradually increasing force (0–50 g) over a 20 s period. If the animal removes their hind paw from the rod, the stimulation stops automatically, and the force and time are recorded.

### 4.11. Tissue Sample Preparation

Either three days or seven weeks after transplantation, animals were perfused with saline and then fixed using 4% paraformaldehyde (PFA, Merck, Darmstadt, Germany). Spinal cords were removed, immersion fixed in 4% PFA for 24 hr at 4 °C, and then cryoprotected using 30% sucrose (Duchefa biochemie, Haarlem, The Netherlands) in PBS for one week at 4 °C. The cryoprotected tissue was embedded in optimal cutting temperature compound (Sakura Finetek, Torrance, CA, USA) and frozen at −80 °C. Tissue sections (20 µm) were then cut using a cryostat.

### 4.12. Histology

#### 4.12.1. Immunohistochemistry

Tissue sections were washed three times using ice-cold 0.3% Tween 20 (Sigma-Aldrich, P1379) in PBS and blocked with 10% normal donkey serum (Jackson ImmunoResearch, West grove, PA, USA) in PBS containing 0.3% Triton X-100 (Sigma-Aldrich, X100) for 1 hr at RT. The blocked sections were incubated overnight with diluted primary antibodies including mouse anti-HuN (1:300; Abcam, Cambridge, MA, USA), mouse anti-CD68 (1:300; Abcam), goat anti-GFAP (1:500, Abcam), chicken anti-MBP (1:400, Abcam), rabbit anti-Tuj1 (1:2000, Abcam), rabbit anti-IL-1β (1:500, Abcam), rabbit anti-NT-3 (1:200, Abcam), and rabbit anti-GDNF (1:100, Abcam) in 10% NDS at 4 °C. After incubations, tissues were washed three times with ice-cold 0.3% Tween 20 and treated with species-specific secondary antibodies, including FITC-donkey anti-mouse IgG (H + L) (1:150, Jackson ImmunoResearch), CyTM3-donkey anti-rabbit IgG (H + L) (1:600, Jackson ImmunoResearch), DyLightTM405-donkey anti-chicken IgY++ (H + L) (1:600, Jackson ImmunoResearch), and CyTM5-donkey anti-goat IgG (H + L) (1:200, Jackson ImmunoResearch) for 1 hr at RT. The slide-mounted tissue was coverslipped using mounting solution with or without DAPI (Vector Laboratories, Inc., Burlingame, CA, USA). Fluorescence imaging was observed and documented using a scanning confocal laser microscope (LSM700, Carl Zeiss, Oberkochen, Germany).

#### 4.12.2. Hematoxylin and Eosin Staining

Tissues were fixed with acetone and air-dried for 10 min. The dried tissue was then rehydrated with PBS for 10 min and stained using hematoxylin (Sigma-Aldrich) for 50 s. After this staining, the tissue was washed using distilled water for 5 min and then stained with eosin (MERCK) for 40 s. The tissue was then dehydrated using graded ethanol solutions (70%, 80%, 90%, and 100%). Permanent mounting medium (Fisher Scientific, Hamptom, NH, USA) was used for tissue coverslipping, and a light microscope (IX71, Olympus, Tokyo, Japan) was used to observe the staining.

#### 4.12.3. Eriochrome Cyanine (EC) Staining

Tissue was fixed in acetone for 10 min and air-dried for 2 hr. The dried tissue was then incubated in EC solution (MERCK, Kenilworth, NJ, USA) for 10 min at RT. After incubation, the tissue was washed with running water and placed in 5% iron alum (MERCK) until the gray matter was distinguishable. Tissues were differentiated using a borax-ferricyanide solution (MERCK) and dehydrated through graded ethanol solutions (70%, 80%, 90%, and 100%). Permanent mounting medium was used for tissue coverslipping, and a light microscope was used to observe the staining.

### 4.13. Statistical Analysis

All data are presented as mean ± standard error of the mean (SEM). The statistical differences between groups were estimated by either a one-way or a two-way analysis of variance (ANOVA) for multiple comparisons. The one-way ANOVA was performed using Tukey’s post-hoc tests and the two-way ANOVA was performed using a mixed-effects model. The *p*-values are indicated as follows: * *p* < 0.05, ** *p* < 0.01, *** *p* < 0.001 and **** *p* < 0.0001. All statistical analyses were performed using GraphPad PRISM 8.3 (GraphPad Software Inc, San Diego, CA, USA).

## Data Availability

The data presented in this study are available in this article.

## Supplementary Materials

The following are available online at https://www.mdpi.com/article/10.3390/ijms22084141/s1, Table S1. The information of antibodies used in this study. Table S2. The information of primers used in this study.
