# Supplementary material for: Peripheral Nerve-Derived Stem Cell Spheroids Induce Functional Recovery and Repair after Spinal Cord Injury in Rodents
Source: Int J Mol Sci. 2021 Apr 16;22(8):4141. doi: 10.3390/ijms22084141 (PMC8072978; doi:10.3390/ijms22084141)
Supplement: Supplementary file 1 [file ijms-22-04141-s001.zip › Supporting_Information_Table_S1_Antibody_Information.docx]

Supporting Information Table S1. The information of antibodies used in this study.

| Antibody | Host | Clonality | Clone name | Dilution fold | Supplier |
| --- | --- | --- | --- | --- | --- |
| A2B5 | Mouse | Monoclonal | A2B5-105 | 1:100 | Millipore |
| CD29 | Rat | Monoclonal | MB1.2 | 1:200 | Millipore |
| CD31 | Rabbit | Polyclonal |  | 1:200 | Abcam |
| CD45 | Mouse | Monoclonal | A20 | 1:500 | Invitrogen |
| CD105 | Mouse | Monoclonal | SN6 | 1:200 | Bio-Rad |
| GFAP | Rabbit | Polyclonal |  | 1:2000 | Dako |
| Tuj1 | Rabbit | Polyclonal |  | 1:500 | Millipore |
| MBP | Mouse | Monoclonal | MBP101 | 1:200 | Abcam |
| Nestin | Mouse | Monoclonal | Rat-401 | 1:300 | Developmental Studies Hydridoma Bank |
| NF200 | Mouse | Monoclonal | 2H3 | 1:1,000 | Developmental Studies Hybridoma Bank |
| P0 | Rabbit | Polyclonal |  | 1:500 | Biorbyt |
| p75^NTR^ | Rabbit | Polyclonal |  | 1:300 | Abcam |
| S100β | Mouse | Monoclonal | SH-B1 | 1:200 | Sigma-Aldrich |
| Sox2 | Rabbit | Polyclonal |  | 1:200 | Millipore |
| Sox9 | Rabbit | Polyclonal |  | 1:1000 | Millipore |
| Sox10 | Goat | Polyclonal |  | 1:200 | R&D Systems |
| HuN | Mouse | Monoclonal |  | 1:300 | Abcam |
| GFAP | Goat | Polyclonal |  | 1:500 | Abcam |
| MBP | Chicken | Polyclonal |  | 1:400 | Abcam |
| Tuj1 | Rabbit | Polyclonal |  | 1:2000 | Abcam |
| IL-1β | Rabbit | Polyclonal |  | 1:500 | Abcam |
| CD68 | Mouse | Monoclonal |  | 1:300 | Abcam |
| NT-3 | Rabbit | Polyclonal |  | 1:200 | Abcam |
| GDNF | Rabbit | Polyclonal |  | 1:100 | Abcam |
| Alexa Fluor^®^ 488 conjugated anti-mouse IgG | Goat | Polyclonal |  | 1:1000 | Invitrogen |
| Alexa Fluor^®^ 488 conjugated anti-rabbit IgG | Goat | Polyclonal |  | 1:1000 | Invitrogen |
| Alexa Fluor^®^ 488 conjugated anti-rat IgG | Goat | Polyclonal |  | 1:1000 | Invitrogen |
