# Supplementary material for: Peripheral Nerve-Derived Stem Cell Spheroids Induce Functional Recovery and Repair after Spinal Cord Injury in Rodents
Source: Int J Mol Sci. 2021 Apr 16;22(8):4141. doi: 10.3390/ijms22084141 (PMC8072978; doi:10.3390/ijms22084141)
Supplement: Supplementary file 1 [file ijms-22-04141-s001.zip › Supporting_Information_Table_S2_Primer_Information.docx]

Supporting Information Table S2. The information of primers used in this study.

| Gene | Sequence (5’ to 3’) | | Size  (bp) | Annealing Temp.(℃) |
| --- | --- | --- | --- | --- |
|  | Forward | Reverse |  |  |
| *BDNF* | GCTGAGCGTGTGTGACAGTA | GGATTGCACTTGGTCTCGTA | 153 | 58 |
| *GDNF* | GCTGAGCAGTGACTCAAAT | CGATTCCGCTCTCTTCTAGG | 142 | 58 |
| *IGF* | CACTTCTTTCTACACAACTCGGGC | CGACTTGCTGCTGCTTTTGAG | 147 | 58 |
| *IL-6* | AGACAGCCACTCACCTCTTCAG | TTCTGCCAGTGCCTCTTTGCTG | 132 | 58 |
| *NGF* | GTGGGTTGGGGATAAGACCA | GCTGTCAACGGGATTTGGGT | 140 | 58 |
| *NT-3* | AAGCTCTCCAAGCAGATGGT | CTCTGTTGTCGCAGCAGTTC | 152 | 58 |
| *GAPDH* | GTGGACCTGACCTGCCGTCT | GGAGGAGTGGGTGTCGCTGT | 134 | 58 |
